# Sleep apnea in patients with exacerbated heart failure and overweight

**DOI:** 10.1016/j.sleepx.2023.100065

**Published:** 2023-02-28

**Authors:** Petar Kalaydzhiev, Nikolay Poroyliev, Desislava Somleva, Radostina Ilieva, Dimitar Markov, Elena Kinova, Asen Goudev

**Affiliations:** a Medical University, Sofia, Bulgaria; b University Hospital “Tsaritsa Yoanna – ISUL”, Byalo More St Ν8, Clinical of Cardiology, Sofia, Bulgaria

**Keywords:** Exacerbated heart failure, Obstructive sleep apnea, Central sleep apnea

## Abstract

Sleep disorders are a common concomitant comorbidity in patients with heart failure. The aims of our study are to determine the incidence and phenotypic characteristics of sleep apnea in overweight patients with exacerbated heart failure and to assess the degree of involvement of systolic and diastolic function impairment in the individual group. From 100 screened patients with heart failure in our department from 2015 to 2017, 61 met the inclusion criteria and participated in the study. 82% (n = 50) of the patients had obstructive sleep apnea (OSA), and 18% (n = 11) had central sleep apnea (CSA). The CSA group had a significantly lower left ventricular ejection fraction (LVEF) than the OSA group (EF% 49.6 ± 8.5 vs 41.8 ± 11.4; p = 0.013). A negative correlation was found between LVEF and the number of central apnea events (r = −0.52; p < 0.001). More frequent hospitalizations for heart failure (HF) and higher mortality rate were found in the CSA group. Screening for sleep apnea in patients with exacerbated heart failure and obesity is necessary for the complex treatment of these patients.

## Highlights

• Obstructive sleep apnea is more common.
• There was no association between AHI and NTproBNP.
• Reduced systolic function is associated with more central sleep apnea events.
• Patients with central sleep apnea and HF have more frequent rehospitalizations.

## 1 Introduction

Sleep apnea is more common in patients with heart failure (HF) than in the general population. There are two main types of sleep apnea. Obstructive sleep apnea is caused by an obstruction of the upper airway, while central sleep apnea is caused by a lack of signal from the central nervous system to the respiratory muscles. The comorbidity rate in patients with OSA and heart failure is high. The frequency varies from 11 to 38% in different studies. In patients with CSA, this percentage is even higher 28–82% [1]. This statistic raises the question of the diagnosis and treatment of these comorbidities. In these patients, hospitalization due to exacerbation of HF is a more frequent phenomenon compared to patients without sleep apnea [2]. Treatment options are being explored, and currently positive results are reported only in the obstructive type, while in the case of central apnea, the studies have not reported definite benefits [3]. The high comorbidity rate may be due to the fact that the two disorders, sleep apnea and heart failure, share common risk factors [4,5]. There is a lack of sufficient data on the type of sleep apnea that prevails during hospitalization for exacerbated heart failure. It is not established whether there is an association between high NTproBNP (N-Terminal Fragment of the Prohormone Brain-Type Natriuretic Peptide - NT-proBNP) and Apnea-Hypopnea Index (AHI) and whether it can be used as a predictive value for the severity of sleep apnea in these patients, which we will seek an answer in the present study. Our study aims to differentiate the frequency and type of sleep apnea in patients with overweight and exacerbated heart failure, evaluate the systolic and diastolic function in the individual groups, and determine whether there is a correlation between the severity of heart failure and the type of sleep apnea.

## 2 Materials and methods

We conducted a single-centre, prospective cohort study in which patients hospitalized in the Cardiology Clinic of UMHAT “Tsaritsa Joanna - ISUL” took part from 2015 to 2017. In 100 consecutive patients with clinical and laboratory evidence of exacerbated heart failure - New York Heart Association (NYHA) class II/III and Body mass index (BMI) > 25 kg/m2, additional laboratory methods were used to assess the degree of heart failure using NTproBNP. Measurements were conducted on a Point of Care - Roche Cobas h 232 System. Patients with NTproBNP values > 300 pg/ml were included. The screening for sleep apnea was conducted using the Epworth Sleepiness Scale (ESS) and an ApneaLink™ somnographic screening system, which was attached to patients on the first night of their stay in the unit. The number and type of apneas and hypopneas per hour were measured - Apnea-Hypopnea Index (AHI). The somnographic recordings were analysed with ApneaLink™ Reporting Software to determine the sleep apnea phenotype. All patients with ESS >6 pts and AHI >5 were included. Two-dimensional (2D) echocardiography was used to assess systolic and diastolic function. To assess systolic function, measurement of the left ventricular ejection fraction using the Simpson method was used, and to measure diastolic function, the ratio E/e'm (the ration of the wave of early diastolic filling of the mitral inflow- E and the tissue Doppler velocity of the medial mitral annulus-e'm) was used. Exclusion criteria were: acute respiratory failure, acute coronary syndrome, severe renal or hepatic failure, and chronic lung diseases (COPD). Sixty-one patients met the inclusion criteria. They were divided into two groups according to the type of sleep apnea - with CSA and with OSA. Patients were followed up for HF hospitalizations and mortality rate over a two-year period. The statistical analysis was performed by SPSS 22.0 (Chicago, Illinois). Statistical methods for comparison using Pearson's chi-squared test and Student's t-test as appropriate. Correlation analysis for linear dependence were used. Simple linear regression was performed to test significantly predicted value. The Kaplan-Meier method was used to analyze the survival rate and first HF hospitalization. The comparison between the two groups was performed using the Log Rank (Mantel-Cox) test. Data with a p-value <0.05 were considered significant.

## 3 Results

From 100 screened consecutive patients, sleep apnea was found in 61% of them (n = 61). Of these, 82% (n = 50) had obstructive sleep apnea, and 18% (n = 11) had central sleep apnea. Regarding the demographic indicators of age and gender, no significant differences were found in the individual groups.

When comparing the ejection fraction (EF%) of the left ventricle between the two groups, significantly lower values of EF were recorded in the group with central sleep apnea compared to the group with OSA (LVEF % 49.6 ± 8.5 vs 41.8 ± 11.4; p = 0.013).

There is also a difference in the diastolic dysfunction indicators, with the E/e'm ratio in the CSA group being significantly higher (E/e'm-17.1 ± 3.7 vs 20.9 ± 2.5; p = 0.002). The NTproBNP values also support this data, patients with CSA have significantly higher values compared to patients with OSA (2857.36 ± 1090.90 pg/ml vs 1359.12 ± 740.64 pg/ml, p = 0.001).

Regarding BMI, significantly higher values were found in the OSA group compared to the patients with CSA (BMI 38.5 ± 7.1 vs 31.9 ± 4.5; p = 0.005).

No significant difference was registered in the degree of severity regarding sleep disorder. Comparison of AHI between the two groups showed no difference (OSA 41.8 ± 23.2 vs CSA 37.7 ± 12.6; p = 0.575). The data summary is presented in Table 1.

**Table 1:** Summary of demographic, echocardiographic and sleep parameters.

	OSA group (n = 50)	CSA group (n = 11)	P Value
Age, yr	66.2 ± 9,1	66.1 ± 11.9	0.991
Sex, m%	m 52%	m 54%	0.878
ESS	12.1 ± 2.9	10,6 ± 3.2	0.144
**LVEF, %**	**49.6** ± **8.5**	**41.8** ± **11.4**	**0.013**
AHI	41.8 ± 23.2	37.7 ± 12.6	0.575
**BMI**	**38.5** ± **7.1**	**31.9** ± **4.5**	**0.005**
**E/e'm**	**17.1** ± **3.7**	**20.9** ± **2.5**	**0.002**
**NTproBNP, pg/ml**	**1359.12** ± **740.64**	**2857.36** ± **1090.9**	**0.001**
Av. Saturation, %	83.9 ± 6.8	86.6 ± 6.6	0.257
Low Saturation, %	65.3 ± 12.7	67.6 ± 12.8	0.590
Av. Pulse, bpm	75.5 ± 11.1	76.4 ± 11.7	0.825
Max. Pulse, bpm	131.1 ± 42.5	121.4 ± 51.2	0.511
N of Desaturation	381.1 ± 212.6	397.5 ± 184.4	0.813

Obstructive sleep apnea(OSA); Central sleep apnea(CSA); male %(m%); Epworth Sleepiness Scale (ESS); Apnea-Hypopnea Index (AHI); The ration of the wave of early diastolic filling of the mitral inflow- E and the tissue Doppler velocity of the medial mitral annulus-e'm(E/e'm); Left ventricular ejection fraction in %(LVEF%); N-Terminal Fragment of the Prohormone Brain-Type Natriuretic Peptide – (NT-proBNP); beats per minute (bpm).

Standard heart failure therapy during the study period included angiotensin converting enzyme inhibitors or angiotensin receptor blockers (ACEi or ARB), Sacubitril/Valsartan, beta blockers and diuretics. Patients in both groups were on standard heart failure therapy. The percentage distribution is shown in Table 2. There was no significant difference in therapy between the two groups.

**Table 2:** Distribution of used medications.

Medication	OSA group (n = 50)	CSA group (n = 11)	P Value
Beta blockers (%)	92%	82%	0.294
ACEi or ARB or Sacubitril/Valsartan (%)	84%	64%	0.133
Mineralocorticoid blockers (%)	70%	82%	0.350
Loop and thiazide diuretics (%)	62%	82%	0.185

Obstructive sleep apnea(OSA); Central sleep apnea(CSA); angiotensin converting enzyme inhibitors (ACEi); angiotensin receptor blockers (ARB).

**Fig. 1:**
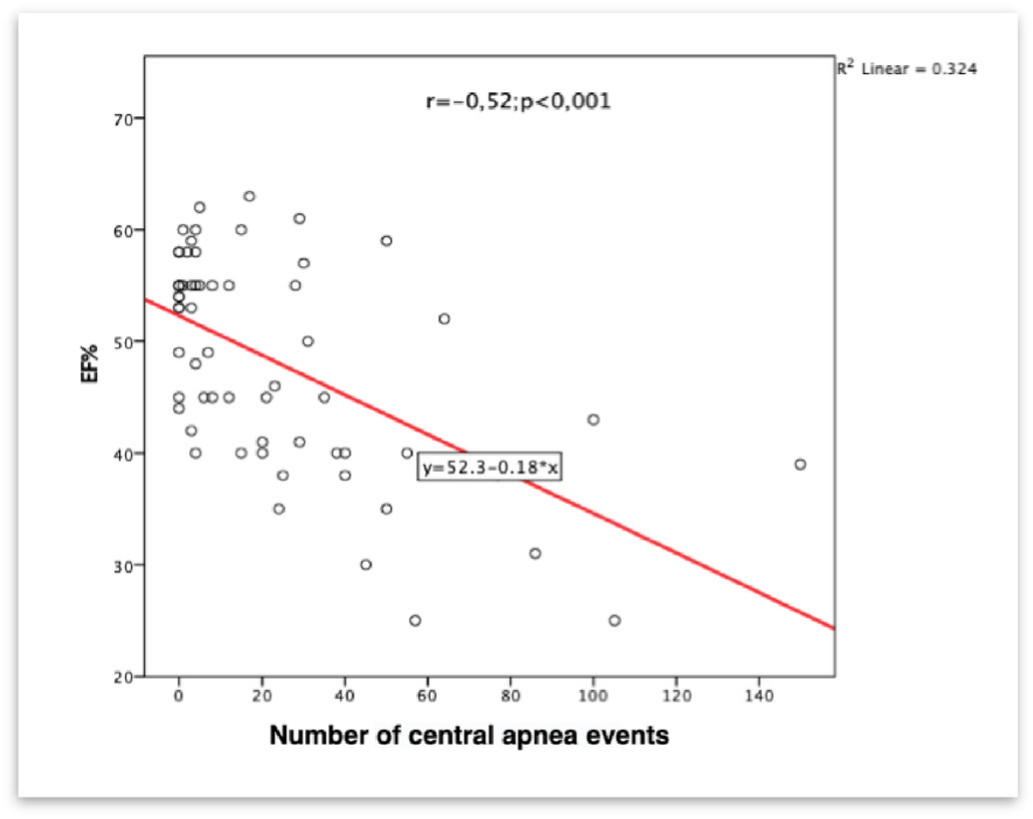
Correlation analysis of the relationship between ejection fraction and number of central sleep apnea events. EF% - ejection fraction %.

After conducting a correlation analysis, a strong negative correlation was found between the number of central apnea events (apneas and hypopneas) and the left ventricular ejection fraction r = −0.52, p < 0.001. The distribution and data are presented in Fig. 1.

Simple linear regression was used to test if the left ventricular ejection fraction significantly predicted the number of central apnea events. The overall regression was statistically significant (R^2^ = 0.32, F(1, 59) = 28.26, p < 0.000). It was found that the left ventricular ejection fraction significantly predicted the number of central apnea events (β = -1.829, p < 0.000).

A strong correlation was also found between BMI and the degree of daytime sleepiness based on the ESS (r = 0.649; p < 0.001). Simple linear regression was used. It was found that the BMI significantly predicted the ESS (β = 0.27, p < 0.000).

No correlation was found between AHI and NTproBNP (r = 0.038; p = 0.770).

Patients from both groups were followed up regarding first hospitalization for heart failure and mortality over a period of 24 months. Mortality in the OSA group for 2 years was 38% (n = 19), and 63.6% (n = 7) in the CSA group.

**Fig. 2:**
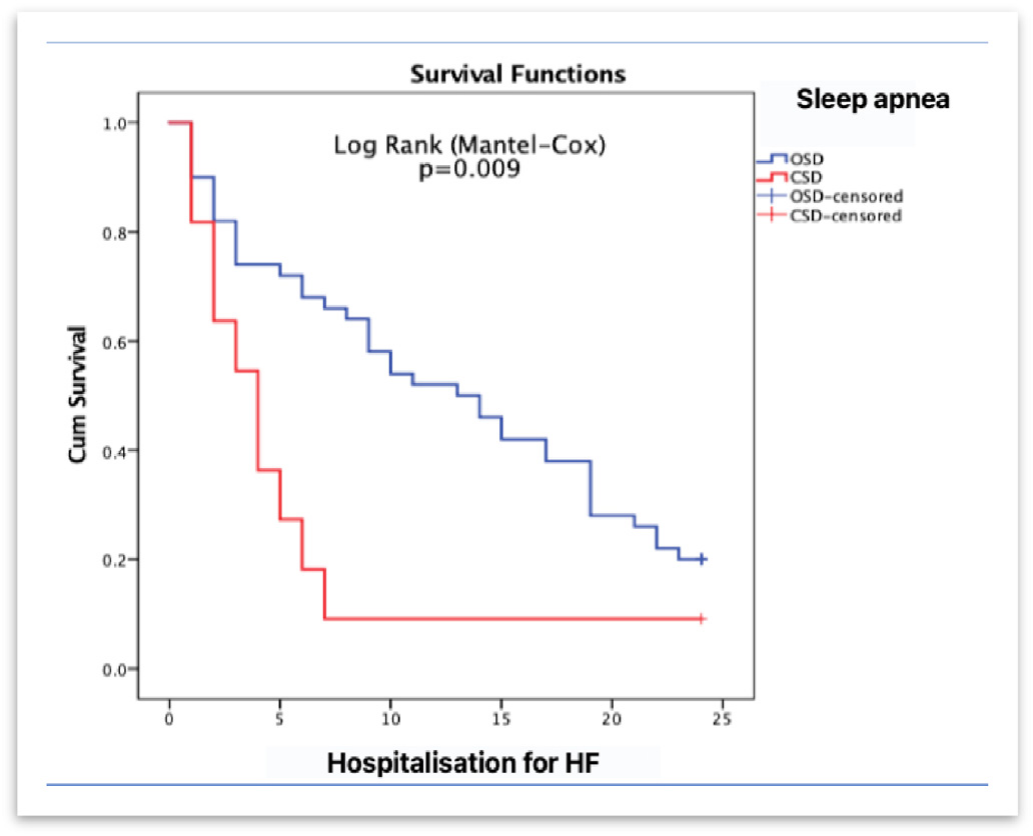
Survival Functions Kaplan-Meier method for time to first hospitalization for Heart Failure (HF) in months Obstructive sleep disease (OSD); Central sleep disease (CSD).

First hospitalization in patients with CSA occurs significantly sooner than in patients with OSA. The average number of months without hospitalization for HF in patients with CSA was 5.3 months versus 12.8 months in patients with OSA (Log Rank (Mental-Cox) p = 0.009). Refer to Fig. 2.

**Fig. 3:**
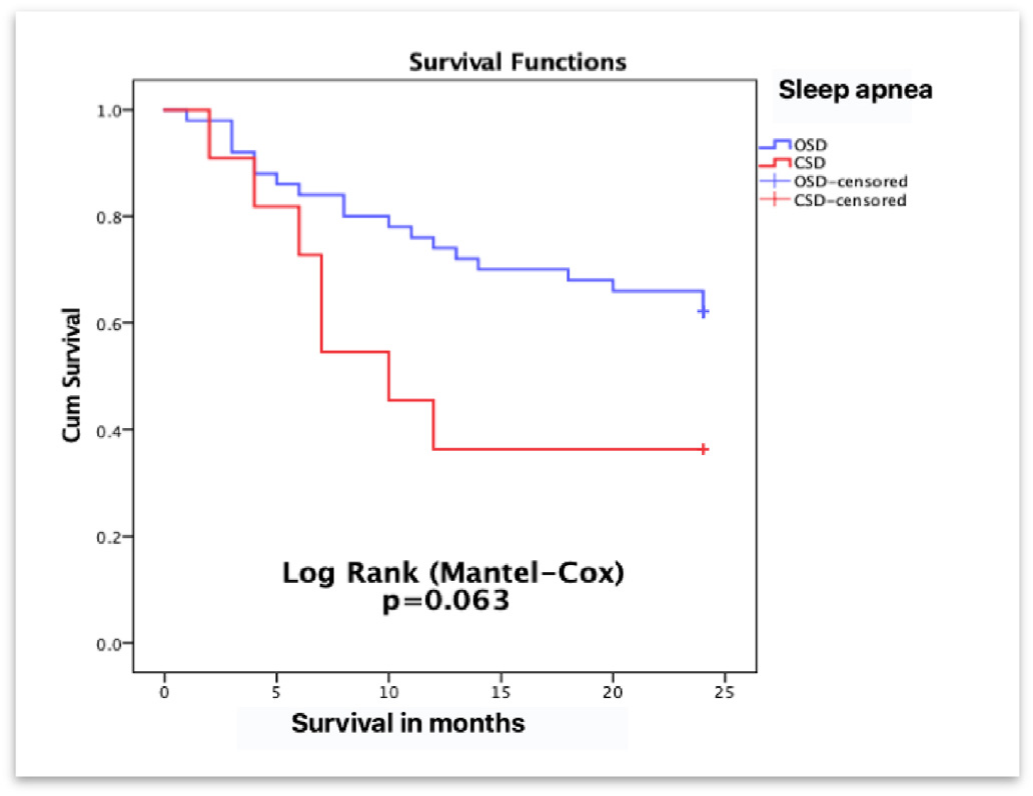
Survival Functions Kaplan-Meier method survival in months. Obstructive sleep disease (OSD); Central sleep disease (CSD).

The OSA group had a median survival of 18.7 months versus 13.09 months in the CSA group. The data approached but did not exceed the limit of significance (p = 0.063). The survival curve is presented in Fig. 3.

## 4 Discussion

For the first time in Bulgaria, a prospective cohort study is being conducted on sleep apnea and exacerbated heart failure and overweight. In our study we found that 61% of tested patients have sleep apnea. 82% (n = 50) of patients had OSA, and 18% (n = 11) had CSA. On a global scale, a study on this topic was conducted by Schulz R et al. [6]. Two hundred and three patients with exacerbated heart failure were studied, of whom 145 (71%) had sleep apnea. The distribution of the OSA and CSA percentage is also at the expense of OSA, without obesity being an inclusion criterion. The percentage of patients with central sleep apnea in the study by Sin and coworkers was significantly higher which may be due to the larger number of patients studied [7]. In 2017 Arzt M et al. performed an extensive analysis of patients in the German registry SchlaHF for patients with reduced systolic function. A high rate of CSA was also found in their study [8]. The high repeatability of the data confirms the need to screen heart failure patients for sleep apnea.

We found significant differences between patients with obstructive sleep apnea and those with central sleep apnea in terms of ejection fraction and diastolic function. Decreased systolic function, elevated NTproBNP values, and increased left ventricular filling pressures, increase the risk of central apnea events. A possible pathophysiological explanation for this phenomenon is that patients with reduced systolic function also have a lower left ventricular filling pressure, as well as increased pulmonary pressure, which increases the risk of hyperventilation and Cheyne-Stokes breathing during sleep [8,9]. We used linear regression analysis to demonstrate the strong correlation between the occurrence of central apneas and decreased systolic function. Left ventricular ejection fraction has a significant predictive value for occurrence of Cheyne-Stokes breathing (R^2^ = 0.32, F(1, 59) = 28.26, p < 0.000).

BMI is a definite risk factor for both SA and heart failure [10,11]. Sin DD et al. studied 450 patients with congestive heart failure, with obesity being the leading risk factor for concomitant sleep disorder. Similar results were published by Lee SJ et al., who found a strong correlation between BMI and daytime sleepiness (ESS) [12]. In our research, we also confirm this dependence.

To support the diagnosis of exacerbated heart failure, we additionally used NTproBNP tests. As expected, they were significantly higher in the CSA group, corresponding to the lower systolic function in these patients. We found no correlation between AHI and NTproBNP. A similar comparison was also conducted by Hübner RH et al. in the study of 60 patients with obstructive sleep apnea and heart failure [13]. This shows us that NTproBNP can't help us with the degree of severity of sleep apnea as well as its type.

In our study, we found that patients with central sleep apnea had significantly more frequent hospitalization for heart failure than patients with obstructive sleep apnea (Log Rank (Mental-Cox) p = 0.009) Fig. 2. This conclusion was also reached by Khayat R et al. in their study [14]. Similar to us, they followed up patients with exacerbated heart failure and sleep disorders, and rehospitalization for heart failure in the CSA group was significantly higher than that in the OSA group. The lower systolic function and higher percentage of hospitalizations in the CSA group determines the worse quality of life in these patients. Although both groups were on standard heart failure therapy, lower systolic function in the CSA group was an independent risk factor for higher rehospitalization and higher mortality [15].

Mortality in the CSA group was higher, approaching but not reaching significance. In another of their publications, Khayat and coworkers discuss the increased mortality rate in patients with CSA and acute heart failure [16].

Timely diagnosis could help the addition of adjunctive therapy to patients with sleep disorders and exacerbated heart failure, which would improve the prognosis especially in patients with low systolic function [17].

One of the major limiting factors in our study is the low number of patients in the CSA group. Analyzing the cohort in more recruited patients would allow the conclusions drawn to be confirmed or rejected. Studies in this field are highly limited due to the multistage conduct of the studies, the hospitalization of the patients, and the severe general condition of exacerbated symptoms of heart failure. Globally, the main studies also have low numbers of patients.

Due to economic constraints, continued positive airway pressure (CPAP) therapy was not added to the patients with OSA, which allowed the comparison between the two groups. Adding CPAP therapy to the management of patients with OSA would undoubtedly improve the prognosis of these patients [18].

Since patient recruitment and follow-up, there have been significant advances in heart failure therapy and the use of new classes of medications such as SGLT2 inhibitors, which in our patients were not included [19]. Which confirms the need for new studies in this area.

## 5 Conclusion

Sleep apnea is a common comorbidity in patients with exacerbated heart failure and obesity. OSA occurs to a greater extent than CSA. Patients with reduced systolic function are at higher risk of central sleep apneas events. Low LVEF% can be used as a prognostic factor regarding the occurrence of central sleep apnea events. Controlling sleep apnea can reduce patient readmissions and mortality. Large-scale, long-term randomized trials will be needed to test the possibility of finding an effective therapy for central sleep apnea would improve the prognosis of patients with exacerbated heart failure and overweight.
